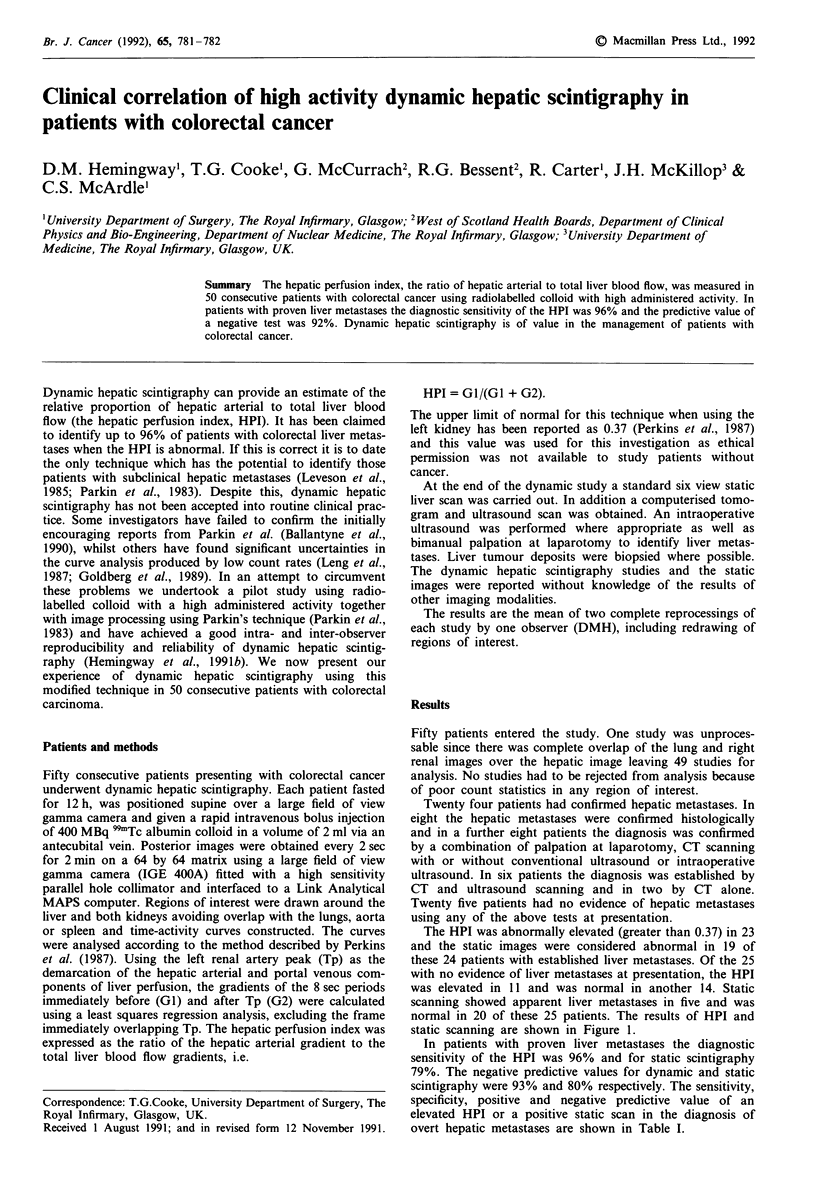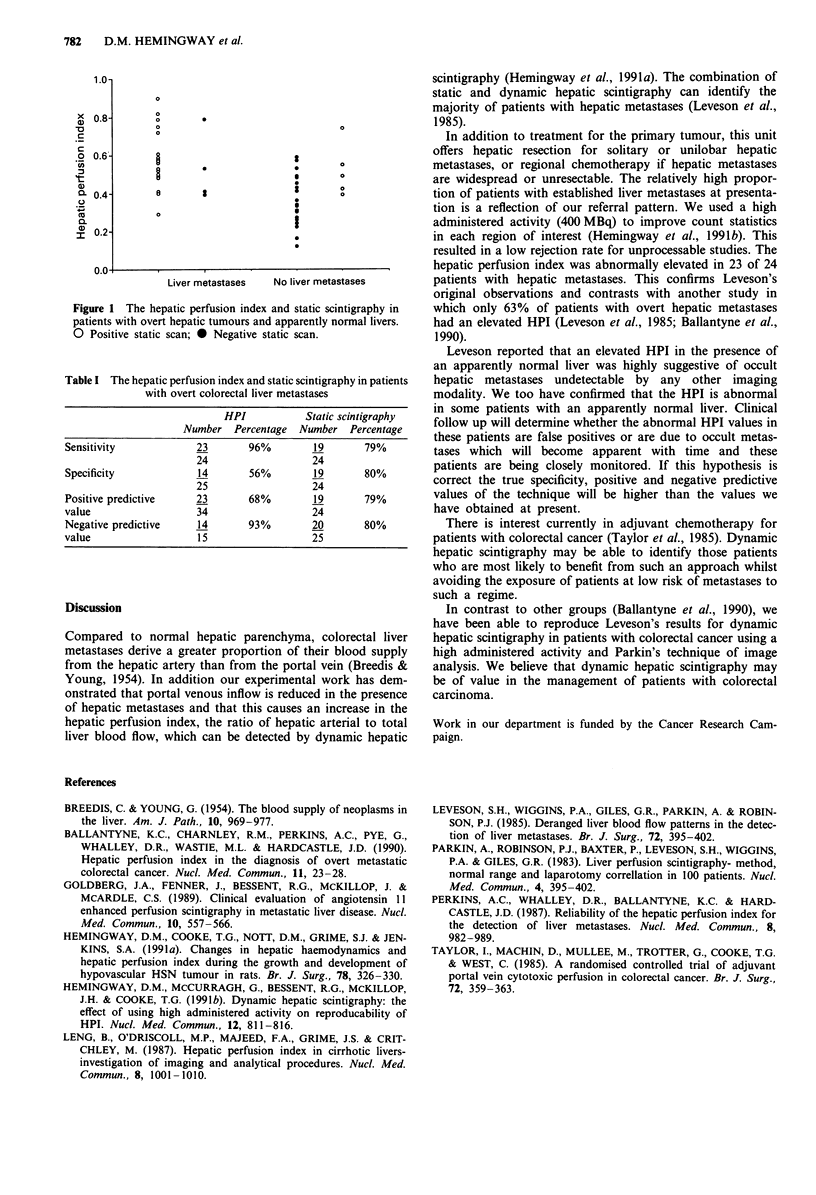# Clinical correlation of high activity dynamic hepatic scintigraphy in patients with colorectal cancer.

**DOI:** 10.1038/bjc.1992.166

**Published:** 1992-05

**Authors:** D. M. Hemingway, T. G. Cooke, G. McCurrach, R. G. Bessent, R. Carter, J. H. McKillop, C. S. McArdle

**Affiliations:** University Department of Surgery, Royal Infirmary, Glasgow, UK.

## Abstract

The hepatic perfusion index, the ratio of hepatic arterial to total liver blood flow, was measured in 50 consecutive patients with colorectal cancer using radiolabelled colloid with high administered activity. In patients with proven liver metastases the diagnostic sensitivity of the HPI was 96% and the predictive value of a negative test was 92%. Dynamic hepatic scintigraphy is of value in the management of patients with colorectal cancer.


Br. J. Cancer (1992), 65, 781-782 © Macmillan Press Ltd., 1992

### Clinical correlation of high activity dynamic hepatic scintigraphy in patients with colorectal cancer

D.M. Hemingway[1], T.G. Cooke[1], G. McCurrach[2], R.G. Bessent[2], R. Carter[1], J.H. McKillop[3] & C.S. McArdle[1]

[1]*University Department of Surgery, The Royal Infirmary, Glasgow;* [2]*West of Scotland Health Boards, Department of Clinical Physics and Bio-Engineering, Department of Nuclear Medicine, The Royal Infirmary, Glasgow;* [3]*University Department of Medicine, The Royal Infirmary, Glasgow, UK.*

**Summary** The hepatic perfusion index, the ratio of hepatic arterial to total liver blood flow, was measured in 50 consecutive patients with colorectal cancer using radiolabelled colloid with high administered activity. In patients with proven liver metastases the diagnostic sensitivity of the HPI was 96% and the predictive value of a negative test was 92%. Dynamic hepatic scintigraphy is of value in the management of patients with colorectal cancer.

Dynamic hepatic scintigraphy can provide an estimate of the relative proportion of hepatic arterial to total liver blood flow (the hepatic perfusion index, HPI). It has been claimed to identify up to 96% of patients with colorectal liver metastases when the HPI is abnormal. If this is correct it is to date the only technique which has the potential to identify those patients with subclinical hepatic metastases (Leveson *et al.*, 1985; Parkin *et al.*, 1983). Despite this, dynamic hepatic scintigraphy has not been accepted into routine clinical practice. Some investigators have failed to confirm the initially encouraging reports from Parkin *et al.* (Ballantyne *et al.*, 1990), whilst others have found significant uncertainties in the curve analysis produced by low count rates (Leng *et al.*, 1987; Goldberg *et al.*, 1989). In an attempt to circumvent these problems we undertook a pilot study using radiolabelled colloid with a high administered activity together with image processing using Parkin's technique (Parkin *et al.*, 1983) and have achieved a good intra- and inter-observer reproducibility and reliability of dynamic hepatic scintigraphy (Hemingway *et al.*, 1991*b*). We now present our experience of dynamic hepatic scintigraphy using this modified technique in 50 consecutive patients with colorectal carcinoma.

### Patients and methods

Fifty consecutive patients presenting with colorectal cancer underwent dynamic hepatic scintigraphy. Each patient fasted for 12 h, was positioned supine over a large field of view gamma camera and given a rapid intravenous bolus injection of 400 MBq $^{99m}$Tc albumin colloid in a volume of 2 ml via an antecubital vein. Posterior images were obtained every 2 sec for 2 min on a 64 by 64 matrix using a large field of view gamma camera (IGE 400A) fitted with a high sensitivity parallel hole collimator and interfaced to a Link Analytical MAPS computer. Regions of interest were drawn around the liver and both kidneys avoiding overlap with the lungs, aorta or spleen and time-activity curves constructed. The curves were analysed according to the method described by Perkins *et al.* (1987). Using the left renal artery peak (Tp) as the demarcation of the hepatic arterial and portal venous components of liver perfusion, the gradients of the 8 sec periods immediately before (G1) and after Tp (G2) were calculated using a least squares regression analysis, excluding the frame immediately overlapping Tp. The hepatic perfusion index was expressed as the ratio of the hepatic arterial gradient to the total liver blood flow gradients, i.e.

$$HPI = G1/(G1 + G2).$$

The upper limit of normal for this technique when using the left kidney has been reported as 0.37 (Perkins *et al.*, 1987) and this value was used for this investigation as ethical permission was not available to study patients without cancer.

At the end of the dynamic study a standard six view static liver scan was carried out. In addition a computerised tomogram and ultrasound scan was obtained. An intraoperative ultrasound was performed where appropriate as well as bimanual palpation at laparotomy to identify liver metastases. Liver tumour deposits were biopsied where possible. The dynamic hepatic scintigraphy studies and the static images were reported without knowledge of the results of other imaging modalities.

The results are the mean of two complete reprocessings of each study by one observer (DMH), including redrawing of regions of interest.

### Results

Fifty patients entered the study. One study was unprocessable since there was complete overlap of the lung and right renal images over the hepatic image leaving 49 studies for analysis. No studies had to be rejected from analysis because of poor count statistics in any region of interest.

Twenty four patients had confirmed hepatic metastases. In eight the hepatic metastases were confirmed histologically and in a further eight patients the diagnosis was confirmed by a combination of palpation at laparotomy, CT scanning with or without conventional ultrasound or intraoperative ultrasound. In six patients the diagnosis was established by CT and ultrasound scanning and in two by CT alone. Twenty five patients had no evidence of hepatic metastases using any of the above tests at presentation.

The HPI was abnormally elevated (greater than 0.37) in 23 and the static images were considered abnormal in 19 of these 24 patients with established liver metastases. Of the 25 with no evidence of liver metastases at presentation, the HPI was elevated in 11 and was normal in another 14. Static scanning showed apparent liver metastases in five and was normal in 20 of these 25 patients. The results of HPI and static scanning are shown in Figure 1.

In patients with proven liver metastases the diagnostic sensitivity of the HPI was 96% and for static scintigraphy 79%. The negative predictive values for dynamic and static scintigraphy were 93% and 80% respectively. The sensitivity, specificity, positive and negative predictive value of an elevated HPI or a positive static scan in the diagnosis of overt hepatic metastases are shown in Table I.

Correspondence: T.G.Cooke, University Department of Surgery, The Royal Infirmary, Glasgow, UK.
Received 1 August 1991; and in revised form 12 November 1991.

**Figure 1** The hepatic perfusion index and static scintigraphy in patients with overt hepatic tumours and apparently normal livers. O Positive static scan; ● Negative static scan.

**Table I** The hepatic perfusion index and static scintigraphy in patients with overt colorectal liver metastases

| | *HPI* | | *Static scintigraphy* | |
|---|---|---|---|---|
| | *Number* | *Percentage* | *Number* | *Percentage* |
| Sensitivity | 23/24 | 96% | 19/24 | 79% |
| Specificity | 14/25 | 56% | 19/24 | 80% |
| Positive predictive value | 23/34 | 68% | 19/24 | 79% |
| Negative predictive value | 14/15 | 93% | 20/25 | 80% |

### Discussion

Compared to normal hepatic parenchyma, colorectal liver metastases derive a greater proportion of their blood supply from the hepatic artery than from the portal vein (Breedis & Young, 1954). In addition our experimental work has demonstrated that portal venous inflow is reduced in the presence of hepatic metastases and that this causes an increase in the hepatic perfusion index, the ratio of hepatic arterial to total liver blood flow, which can be detected by dynamic hepatic scintigraphy (Hemingway *et al.*, 1991*a*). The combination of static and dynamic hepatic scintigraphy can identify the majority of patients with hepatic metastases (Leveson *et al.*, 1985).

In addition to treatment for the primary tumour, this unit offers hepatic resection for solitary or unilobar hepatic metastases, or regional chemotherapy if hepatic metastases are widespread or unresectable. The relatively high proportion of patients with established liver metastases at presentation is a reflection of our referral pattern. We used a high administered activity (400 MBq) to improve count statistics in each region of interest (Hemingway *et al.*, 1991*b*). This resulted in a low rejection rate for unprocessable studies. The hepatic perfusion index was abnormally elevated in 23 of 24 patients with hepatic metastases. This confirms Leveson's original observations and contrasts with another study in which only 63% of patients with overt hepatic metastases had an elevated HPI (Leveson *et al.*, 1985; Ballantyne *et al.*, 1990).

Leveson reported that an elevated HPI in the presence of an apparently normal liver was highly suggestive of occult hepatic metastases undetectable by any other imaging modality. We too have confirmed that the HPI is abnormal in some patients with an apparently normal liver. Clinical follow up will determine whether the abnormal HPI values in these patients are false positives or are due to occult metastases which will become apparent with time and these patients are being closely monitored. If this hypothesis is correct the true specificity, positive and negative predictive values of the technique will be higher than the values we have obtained at present.

There is interest currently in adjuvant chemotherapy for patients with colorectal cancer (Taylor *et al.*, 1985). Dynamic hepatic scintigraphy may be able to identify those patients who are most likely to benefit from such an approach whilst avoiding the exposure of patients at low risk of metastases to such a regime.

In contrast to other groups (Ballantyne *et al.*, 1990), we have been able to reproduce Leveson's results for dynamic hepatic scintigraphy in patients with colorectal cancer using a high administered activity and Parkin's technique of image analysis. We believe that dynamic hepatic scintigraphy may be of value in the management of patients with colorectal carcinoma.

Work in our department is funded by the Cancer Research Campaign.

### References

BREEDIS, C. & YOUNG, G. (1954). The blood supply of neoplasms in the liver. *Am. J. Path.*, **10**, 969–977.

BALLANTYNE, K.C., CHARNLEY, R.M., PERKINS, A.C., PYE, G., WHALLEY, D.R., WASTIE, M.L. & HARDCASTLE, J.D. (1990). Hepatic perfusion index in the diagnosis of overt metastatic colorectal cancer. *Nucl. Med. Commun.*, **11**, 23–28.

GOLDBERG, J.A., FENNER, J., BESSENT, R.G., MCKILLOP, J. & MCARDLE, C.S. (1989). Clinical evaluation of angiotensin 11 enhanced perfusion scintigraphy in metastatic liver disease. *Nucl. Med. Commun.*, **10**, 557–566.

HEMINGWAY, D.M., COOKE, T.G., NOTT, D.M., GRIME, S.J. & JENKINS, S.A. (1991*a*). Changes in hepatic haemodynamics and hepatic perfusion index during the growth and development of hypovascular HSN tumour in rats. *Br. J. Surg.*, **78**, 326–330.

HEMINGWAY, D.M., MCCURRAGH, G., BESSENT, R.G., MCKILLOP, J.H. & COOKE, T.G. (1991*b*). Dynamic hepatic scintigraphy: the effect of using high administered activity on reproducability of HPI. *Nucl. Med. Commun.*, **12**, 811–816.

LENG, B., O'DRISCOLL, M.P., MAJEED, F.A., GRIME, J.S. & CRITCHLEY, M. (1987). Hepatic perfusion index in cirrhotic livers-investigation of imaging and analytical procedures. *Nucl. Med. Commun.*, **8**, 1001–1010.

LEVESON, S.H., WIGGINS, P.A., GILES, G.R., PARKIN, A. & ROBINSON, P.J. (1985). Deranged liver blood flow patterns in the detection of liver metastases. *Br. J. Surg.*, **72**, 395–402.

PARKIN, A., ROBINSON, P.J., BAXTER, P., LEVESON, S.H., WIGGINS, P.A. & GILES, G.R. (1983). Liver perfusion scintigraphy- method, normal range and laparotomy correllation in 100 patients. *Nucl. Med. Commun.*, **4**, 395–402.

PERKINS, A.C., WHALLEY, D.R., BALLANTYNE, K.C. & HARDCASTLE, J.D. (1987). Reliability of the hepatic perfusion index for the detection of liver metastases. *Nucl. Med. Commun.*, **8**, 982–989.

TAYLOR, I., MACHIN, D., MULLEE, M., TROTTER, G., COOKE, T.G. & WEST, C. (1985). A randomised controlled trial of adjuvant portal vein cytotoxic perfusion in colorectal cancer. *Br. J. Surg.*, **72**, 359–363.